# The Efficacy of Single-Dose versus Double-Dose Praziquantel Treatments on* Schistosoma mansoni* Infections: Its Implication on Undernutrition and Anaemia among Primary Schoolchildren in Two On-Shore Communities, Northwestern Tanzania

**DOI:** 10.1155/2017/7035025

**Published:** 2017-09-28

**Authors:** David Z. Munisi, Joram Buza, Emmanuel A. Mpolya, Teckla Angelo, Safari M. Kinung'hi

**Affiliations:** ^1^Department of Global Health and Bio-Medical Sciences, School of Life Sciences and Bio-Engineering, Nelson Mandela African Institution of Science and Technology (NM-AIST), P.O. Box 447, Arusha, Tanzania; ^2^Department of Bio-Medical Sciences, School of Medicine and Dentistry, College of Health Sciences, University of Dodoma, P.O. Box 259, Dodoma, Tanzania; ^3^National Institute for Medical Research (NIMR), Mwanza Research Centre, Isamilo Road, P.O. Box 1462, Mwanza, Tanzania

## Abstract

Administering more than one treatment may increase Praziquantel cure and egg reduction rates, thereby hastening achievement of schistosomiasis transmission control. A total of 431* S. mansoni*-infected schoolchildren were randomized to receive either a single or repeated 40 mg/kg Praziquantel dose. Heights, weights, and haemoglobin levels were determined using a stadiometer, weighing scale, and HemoCue, respectively. At 8 weeks, cure rate was higher on repeated dose (93.10%) compared to single dose (68.68%) (*p* < 0.001). The egg reduction rate was higher on repeated dose (97.54%) compared to single dose (87.27%) (*p* = 0.0062). Geometric mean egg intensity was lower among those on repeated dose (1.30 epg) compared to single dose (3.18 epg) (*p* = 0.036) but not at 5 (*p* > 0.05) and 8 (*p* > 0.05) months with no difference in reinfection rate. No difference in the prevalence of stunting was observed between the two treatment regimens (*p* > 0.05) at 8 months, but there was an increase in the prevalence of wasting among those on repeated dose (*p* < 0.001). There was an increase in the mean haemoglobin levels at 8 months with no difference between the two arms (*p* > 0.05). To achieve reduction of transmission intensity and disease control in highly endemic areas, repeated treatments alone may not be sufficient. This trial was registered with PACTR201601001416338.

## 1. Introduction

Schistosomiasis transmitted by fresh water snails is one of the highly prevalent parasitic infections in the world, and it is estimated that more than 200 million individuals are infected at any given time, of whom over a half suffer from related morbidity and about 93% are inhabitants of sub-Saharan Africa [1–3]. The disease is responsible for causing considerable morbidity and mortality in endemic rural communities, inflicting up to 4.5 million disability-adjusted life years (DALYs) [4, 5].

Three endemic species, namely,* Schistosoma mansoni*,* S. haematobium*, and* S. intercalatum*, are responsible for causing schistosomiasis in Africa, of which the most important ones are* S. mansoni* and* S. haematobium* that cause intestinal and urinary schistosomiasis, respectively [6–8]. Both intestinal and urinary schistosomiases are major public health problems in Tanzania, where levels of endemicity vary from place to place [9]. In 2012, it was reported that, of the estimated population of 43.5 million people, nearly 23.2 million were infected with schistosomiasis, forming country prevalence of about 51.5%, making the country rank second to Nigeria in terms of disease burden in Africa [7, 9, 10]. In particular,* Schistosoma mansoni* in the country is extensively distributed in the southeastern and southwestern sides of Lake Victoria and its islands [9, 11]. In these areas, it has been reported to significantly affect people, mostly schoolchildren, contributing significantly to their morbidities and mortalities [12–15].

Praziquantel chemotherapy has been the mainstay for schistosomiasis control in many endemic countries [16]. The target of schistosomiasis Praziquantel mass chemotherapy in endemic countries has been to control morbidity associated with the disease and, in certain epidemiological settings, contribute to sustained reduction in transmission of the disease [5, 17, 18]. In these areas, treatment is implemented at periodic intervals, as part of either school-based or community-based campaigns, referred to as mass drug administration (MDA) [19]. However, in 2012, through World Health Assembly Resolution 65.19, WHO recommended that countries, if possible, aim beyond control of morbidity toward elimination of schistosomiasis as also stated in the sustainable development goals for all neglected tropical diseases [20, 21].

Praziquantel has been shown to have good efficacy in killing both mature worms [22]. However, the use of a single dose 40 mg/kg has limitations as PZQ does not kill immature worms present in the body at the time of treatment [22, 23]. When Praziquantel is used in the first dose, it will kill the adult stages only, and in endemic areas chances of having developing immature stages are quite high and these ones are not going to be killed by the first dose; instead, as they mature, they are likely to be exposed to sublethal doses of Praziquantel, therefore increasing chances of developing resistance. In that case, application of a second Praziquantel dose at weeks 3–6 will kill those parasites that were immature during the first dose as they will have matured by then; therefore, in doing so, there will be an improvement in the cure rates and egg reduction rate which in turn will slow down the likelihood of the parasite developing resistance to the drug as well as significantly reducing environmental contamination by eggs discharged by infected people and therefore contributing to the efforts of achieving transmission control [22–26].

This restricted activity to adult worms and eggs may contribute to reduced efficacy of Praziquantel and also contribute to raising population of adult parasites that have once been exposed to the drug and possibly contribute to emergency of Praziquantel resistance [22]. This speculation is supported by studies elsewhere which have reported reduced sensitivity of* Schistosoma mansoni* to Praziquantel and a failure of complete cure in a* S. mansoni* infection with a standard dose [25, 27–29]. In Tanzania, a study done in Mara region showed that even a single Praziquantel treatment could produce a genetic bottleneck with reductions in a range of measures of genetic diversity of Schistosoma* mansoni*; reduction in genetic diversity may be an initial sign of emerging resistance or reduced sensitivity to the drug [30]. Because of this, investigating alternative treatment strategies that may help prolong the usefulness of the drug such as administering multiple doses is highly important [28, 31, 32]. In addition, administering more than one treatment may increase cure rate, thereby significantly hastening efforts to achieve transmission control by 2030 as stated in the sustainable development goals [21]. Therefore this study intended to investigate the efficacy of single and repeated dose Praziquantel treatments on* S. mansoni* infection and its implication on the burden of undernutrition and anaemia among primary schoolchildren living in an endemic area in Rorya District, northwestern Tanzania.

## 2. Methodology

### 2.1. Study Design and Population

This study was done in Rorya District, northwestern Tanzania, from 2015 to 2016. The district forms one of the seven districts that constitute the Mara region. It borders Tarime District to the east, Lake Victoria to the west, Butiama District to the south, and the Republic of Kenya to the north [33]. The Luo tribe constitutes the majority of inhabitants of Rorya District. Other ethnic groups are Kurya, Kine, Simbiti, Sweta, and Suba. The district is situated in the north of Tanzania and lies within latitudes 1°00′′–1°45′′ south of the Equator and longitudes 33°30′′–35°0′′ east of Greenwich Meridian. A more detailed description of the study area is found in our previous publication [12]. The current study was a longitudinal randomized intervention trial with a registration number PACTR201601001416338 registered on the Pan African Clinical Trials Registry. The longitudinal randomized intervention trial aimed at comparing the efficacy of single 40 mg/kg dose against repeated 40 mg/kg dose of Praziquantel treatment regimens on parasitological (egg reduction rate and cure rates) and morbidity (haemoglobin and nutritional status) indicators with cure rate being the primary outcome of interest.

The study population consisted of primary schoolchildren aged 6−16 years attending primary schools in two villages, Busanga and Kibuyi, in Rorya District. The inclusion and exclusion criteria were as described in Munisi et al.'s work [34].

### 2.2. Sample Size Determination

This study was a longitudinal intervention trial that aimed at comparing parasitological cure rates of single versus repeated doses of Praziquantel treatments for the treatment of intestinal schistosomiasis. We used a formula for calculating sample size aimed at comparing two rates to calculate the sample size for this study [35]. Parasitological cure rates of Praziquantel against intestinal schistosomiasis reported from a study of communities living along the shores of Lake Albert in Uganda, which reported cure rates of 41.9% and 69.1% for the single-dose and two-dose treatment regimens, respectively, were used [36]. The level of significance was set at 95% and the study power was set at 90%. We added 30% to counter annual loss to follow-up; a total sample size of 257 schoolchildren was required per treatment group. The sampling procedure was as described in our previous publication [34].

### 2.3. Data Collection

#### 2.3.1. Assessment for Demographic Characteristics

A pretested Swahili translated semistructured interview questionnaire was used to gather demographic information about the study participants. Variables such as age, sex, and sociodemographic characteristics were assessed. Initially, the questionnaire was developed in English; it was then translated to Swahili and back-translated to English by a different person who was blinded to the original questionnaire.

#### 2.3.2. Stool Sample Collection and Examination

Stool containers and wooden applicator sticks were provided to children with signed informed consent forms from their parents or legal guardians; the children were then requested to bring sizable stool samples of their own. We collected a single stool sample from each study participant. To increase sensitivity, a standard protocol with four Kato-Katz thick smears was prepared from different parts of the single stool sample using a template of 41.7 mg (Vestergaard Frandsen, Lausanne, Switzerland) [5, 37, 38]. Examinations of Kato smears for hookworm eggs were performed within 1 hour of slide preparation. Then the Kato smears were arranged in wooden slide boxes, packed together in large container boxes, and transported to the National Institute for Medical Research (NIMR) laboratory, Mwanza Research Centre, where they were examined for* S. mansoni* eggs by two experienced laboratory technicians. The intensity (eggs per gram (epg) of faeces of* S. mansoni* infection for each child) was calculated as an average egg per gram of faeces for all the four Kato smears prepared for each child. We used a template delivering 41.7 mg of stool to prepare Kato slides, the eggs of each parasite in the slide were counted, and the number of eggs was multiplied by 24 to calculate epg for* S. mansoni *infection.* Schistosoma mansoni* intensities were categorized as per WHO intensity classes as light (1–99 epg), moderate (100–399 epg), and heavy (≥400 epg) [5]. A random sample of 10% of the negative and positive Kato-Katz thick smears was reexamined by a third technician as a quality assurance procedure.

#### 2.3.3. Anthropometric Measurements

The children's heights and weights were measured using a portable stadiometer and digital weighing scale, respectively. The children's barefoot stature and weight with minimum clothing and without shoes were recorded to the nearest 0.1 cm and 0.1 kg, respectively. The resulting height and weight measurements were used to calculate *z*-scores using the new World Health Organization's Child Growth Standards [39]. Children with height-for-age *z*-scores (HAZ) and BMI-for-age *z*-scores (BMIAZ) below or equal to −2 standard deviation (≤−2 SD) were classified as stunted and wasted, respectively. Those children whose HAZ and BMIAZ were less or equal to −3 standard deviation (≤−3 SD) were classified as severely stunted and severely wasted, respectively. Body mass index (BMI) was used as the index of choice for the assessment of recent undernutrition as recommended [40]. We took all anthropometric measurements with instruments that were calibrated and validated before use.

The age of each participant was recorded from school records as reported by parents/guardians during school registration of the children. We used the midpoint of the year of birth and the 15th day of the month of birth.

#### 2.3.4. Determination of Haemoglobin Levels

About 100 *μ*l of blood was collected by finger prick using disposable lancet; this was used to determine venous haemoglobin (Hb) by using a HemoCue photometer (HemoCue, Angelholm, Sweden) [41]. Children with Hb levels more than or equal to 11 g/dL were considered to be normal. Anaemia was defined as haemoglobin levels of less than 11 g/dL, while haemoglobin levels of less than 7 g/dL, 7.0–9.9 g/dL, and 10.0–10.9 g/dL were classified as severe anaemia, moderate anaemia, and mild anaemia, respectively [42].

#### 2.3.5. Randomization and Treatment

For assignment to the type of treatment regimen, children with positive stool test for* Schistosoma mansoni* were randomly divided into two groups using SPSS-generated random numbers after entering all collected data from the baseline survey. One group did not receive a second dose of 40 mg/kg PZQ; therefore this was the single treatment arm, that is, only treated at baseline. The second group was assigned to receive a second dose of 40 mg/kg of body weight PZQ with a 3-week interval. Treatment was given using tablets of PZQ USP 600 mg manufactured by Micro Labs Ltd., Verna, Goa, India. The tablets were swallowed under the supervision of a qualified nurse involved in the study.

### 2.4. Data Analysis

We created a database of the collected data using EpiData version 3.1. Then the data were analysed using STATA version 12.1 (Stata Corp, Texas, USA). In the descriptive analysis, we used simple frequency and percentages.

The cure rate was defined as the proportion of treated persons who were egg-positive at baseline but became egg-negative 8 weeks after baseline treatment. We used two-sample proportion comparison test to compare cure rates between the two treatment regimens among different demographic characteristics. The egg reduction rate for those who remained positive was calculated as follows: (1 − [AMI after treatment/AMI before treatment]) × 100. The chi-square test and Fisher's exact test were used to compare proportions of anaemia, stunting, and wasting between the two treatment regimens for different demographic characteristics. Parasite counts were normalized by log transformation, averaged, and then back-transformed to the original scale.* S. mansoni* infection intensities were calculated as geometric mean of eggs per gram of faeces. A *p* value of less than 0.05 was considered as statistically significant.

### 2.5. Ethical Statement

The study was approved by the Medical Research Coordination Committee (MRCC) of the National Institute for Medical Research (NIMR), Tanzania (reference number: NIMR/HQ/R.8a/Vol. IX/1990). The study received further clearance from the District Executive Director, District Education Officer, and District Medical Officer of the Rorya District Council. Before commencement of the study, the research team conducted meetings with the village executive officers, teachers, and pupils of selected villages and schools, respectively. During these meetings, the objectives of the study, the study procedures to be followed, samples to be taken, study benefits, and potential risks and discomforts were explained. Informed consent for all children who participated in the study was sought from parents and legal guardians by signing an informed consent form. Assent was sought from children who were also informed of their right to refuse to participate in the study and to withdraw from the study at any time during the study. At baseline, all children were given a standard dose of Praziquantel (40 mg/kg) and albendazole (400 mg) as a single dose on separate days. Treatment with Praziquantel was given after a meal which was prepared and offered at school to minimize potential side effects. Treatment was performed immediately after baseline data collection and was done under direct observation (DOT) by a qualified nurse.

## 3. Results

### 3.1. Baseline Characteristics of Study Participants and Trial Profile

**Figure 1:**
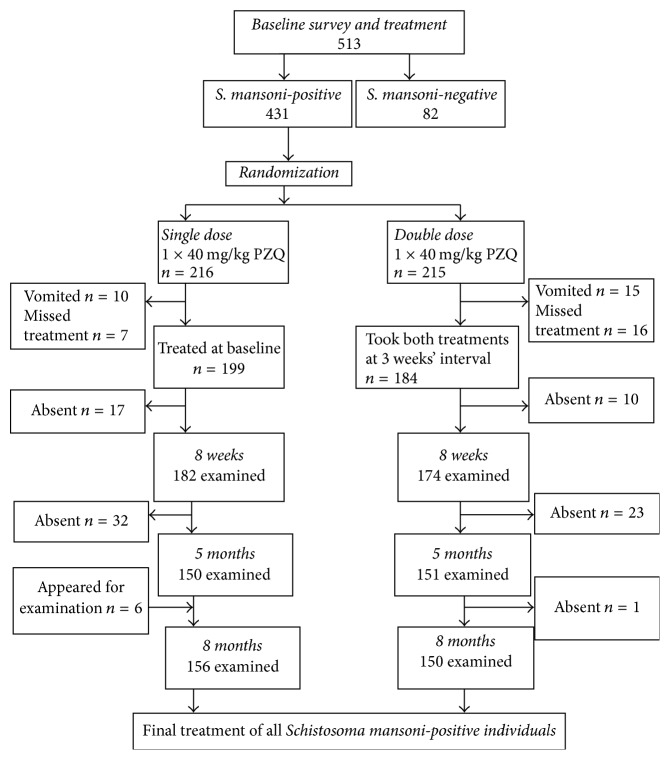
Study profile and compliance among 431* S. mansoni*-infected schoolchildren in an endemic area, northwestern Tanzania.

During the baseline study, we were able to recruit 256 schoolchildren for the single-dose treatment group and 257 for the double-dose treatment group (a total of 513 schoolchildren for the whole study).  Figure 1 shows the trial profile and compliance among study participants. A total of 431 schoolchildren were found to be infected with* Schistosoma mansoni* and were included in the trial. However, upon randomization into the two treatment arms, 199 infected schoolchildren received a single 40 mg/kg Praziquantel dose and 184 infected schoolchildren received two Praziquantel treatments three weeks apart.

Cure rate and egg reduction rate were assessed at 8 weeks after the first treatment and reinfection was assessed 5 and 8 months after the first treatment (baseline treatment).  Table 1 shows baseline characteristics compared between individuals assigned to either of the two treatment arms. Characteristics of individuals were similar with regard to sex distribution, mean age, mean haemoglobin level, mean height, and mean weight but they differed in the baseline geometric mean egg intensity, in which case infected children assigned to receive the two doses of Praziquantel had significantly higher geometric mean egg intensity (GMI) (*p* = 0.0352) as well as arithmetic mean egg intensity (AMI) (*p* = 0.047).

### 3.2. Cure Rate

Table 2 shows the cure rate according to sex, village of residence, and age group of study participants on a single dose of PZQ (40 mg/kg PZQ) compared to two doses of PZQ (2 × 40 mg/kg PZQ) three weeks apart, at 8 weeks after baseline treatment. A significant difference in cure rate was observed between the two treatment regimes, whereby the cure rate among infected schoolchildren who received two doses of Praziquantel treatment (93.10%) was significantly higher compared to that among those who received a single dose of Praziquantel treatment (68.68%) assessed 8 weeks following baseline treatment (*p* < 0.001). This difference was still maintained when cure rates between the two treatment arms were compared for males, females, and village of residence, whereby in all cases cure rates were significantly higher among children who received two doses of Praziquantel treatment (*p* < 0.05). However, when cure rates were compared among subjects in different age groups, a significant difference in cure rates was observed among children belonging to age groups of 6–9 years and 10–12 years only. No significant difference in cure rates was observed among children aged 13–16 years who received single dose and two doses of Praziquantel treatment (*p* = 0.080) (Table 2).

Cure rates were analysed by baseline infection intensity category for the two treatment arms. It was observed that cure rates were significantly higher (*p* < 0.05) among children who received double-dose treatment than among those who received single-dose treatment among children with light, moderate, and heavy intensity infections (Table 2).

### 3.3. Impact of Single Dose versus Double Dose of Praziquantel Treatment on Intensity of* Schistosoma mansoni* Infection

**Figure 2:**
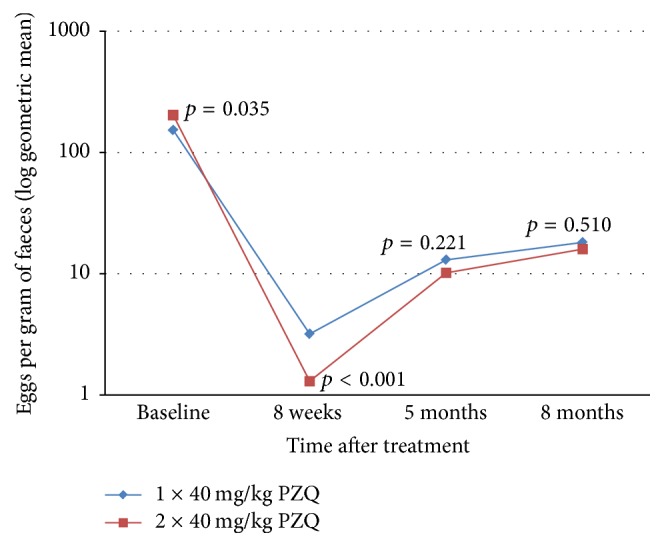
Infection intensity expressed as geometric mean of the log of fecal eggs count per gram of faeces at baseline, 8 weeks, 5 months, and 8 months following treatment of* S. mansoni* infections with a single dose of PZQ (40 mg/kg) versus 2 × 40 mg/kg in the study area.

The effect of the two treatment regimens on reduction of mean egg counts among schoolchildren who were found to be egg-positive at 8 weeks, 5 months, and 8 months after treatment is summarised in Figure 2. At baseline, there was a significant difference in the geometric mean egg intensity per gram of faeces, with children on the double-dose treatment arm bearing higher geometric mean egg count (203.00 epg) than those in the single-dose treatment arm (152.98 epg) (*p* = 0.0352). At 8 weeks following baseline treatment, the geometric mean egg count was particularly low among children who received 2 doses of PZQ (1.30 epg) compared to those who received only one treatment dose (3.18 epg) and the deference was significant. It was further observed that geometric mean egg intensity of* S. mansoni* started to rise after a sharp decline at 8 weeks following baseline treatment. The geometric mean egg count at 5 months after baseline was slightly higher on the single-dose arm (13.03 epg) compared to double-dose arm (10.18 epg) but the difference was not statistically significant (*p* > 0.05). Likewise, the geometric mean egg count at 8 months after baseline was again slightly higher on the single-dose arm (18.14 epg) compared to double-dose arm (15.94 epg) but again the difference was not statistically significant (*p* > 0.05) (Figure 2).

Egg reduction rate is the proportional reduction in number of* S. mansoni* eggs in stool samples. The baseline arithmetic mean egg intensity (AMI) of individuals in the single-dose arm was 344.71 epg (95% CI: 261.13–428.30) and it reduced to 43.88 epg (95% CI: 11.33–76.43) following treatment, resulting in an egg reduction rate of 87.27% (95% CI: 79.93–92.89), while the AMI of individuals in the double-dose arm reduced from 456.29 epg (95% CI: 341.32–571.26) to 11.24 epg (95% CI: 3.27–25.75), resulting in an egg reduction rate of 97.54% (95% CI: 92.96–99.76), and the difference between these two egg reduction rates was statistically significant (*p* = 0.0062).

### 3.4. Reinfection with* S. mansoni* at 5 Months and 8 Months after Treatment with a Single Dose of Praziquantel versus Double Dose of Praziquantel

Reinfection is defined as those people who were positive for* Schistosoma mansoni* at baseline before treatment and became egg-negative at 8 weeks following treatment but later became reinfected. At 5 months after baseline treatment, 74/112 (66.07%) people in the single-dose treatment arm were reinfected, while 102/150 (68.00%) in the double-dose treatment arm were reinfected at this time. These rates of reinfection increased to 100/121 (82.64%) and 114/148 (77.03) in the single-dose and double-dose treatment arms, respectively, at 8 months following baseline treatment. The overall prevalence of reinfection was not statistically significantly different between the two treatment groups at 5 months (*p* = 0.742) and 8 months (*p* = 0.256). After stratification by sex, age, and village of residence, there was only a statistically significant difference in the prevalence of reinfection between the two treatment groups among schoolchildren at Kibuyi village at 8 months after baseline treatment, whereby children who were treated with a single dose of Praziquantel treatment had significantly higher prevalence of reinfection (61/67 (91.04%)) as compared to those who were treated with two doses of Praziquantel three weeks apart (59/78 (75.64)) (*p* < 0.05) (Table 3).

### 3.5. Impact of Single-Dose versus Double-Dose Praziquantel Treatment of* Schistosoma mansoni* Infections on Nutritional Status

The prevalence of stunting at baseline, 40.21% (95% CI: 35.30%–45.12%), was compared with that 8 months after treatment, 36.31% (95% CI: 31.17%–41.45%); although there was a slight decline in the overall prevalence, the difference was not statistically significant (*p* = 0.2833). Prevalence of stunting at baseline and that at 8 months after treatment were compared among individuals who received a single dose of Praziquantel and those who received two doses of Praziquantel three weeks apart. Although in both treatment arms there was a slight decrease of the prevalence of stunting, more so among children on two Praziquantel doses, the difference was not statistically significant (*p* > 0.05) (Table 4).

Again the prevalence of wasting at baseline, 14.10% (95% CI: 10.61%–17.59%), was compared with that 8 months after treatment, 24.40% (95% CI: 19.81%–28.99%). It was observed that, generally, the prevalence of wasting was significantly higher at 8 months after treatment than how it was at baseline (*p* < 0.001). The prevalence of wasting at baseline and that at 8 months after treatment were further compared among individuals who received a single dose of Praziquantel and those who received two doses of Praziquantel three weeks apart. Although the prevalence of wasting was observed to be higher at 8 months after treatment, it was significantly so only among children who received two doses of Praziquantel (*p* < 0.001) (Table 4).

### 3.6. Impact of Single-Dose versus Double-Dose Praziquantel Treatment of* S. mansoni* Infections on Haemoglobin Levels

**Figure 3:**
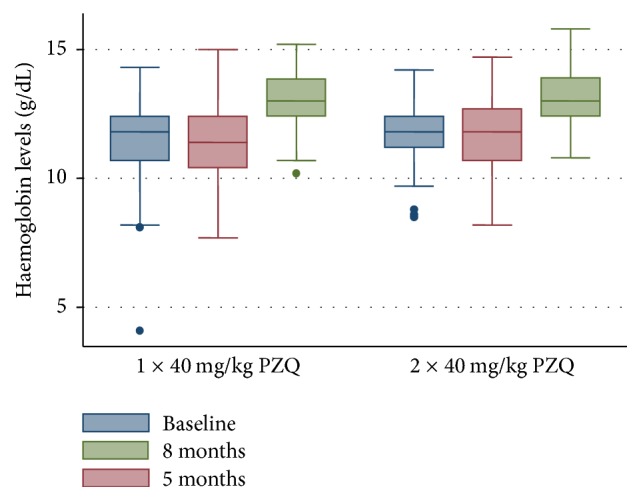
Box-and-whisker plot showing the relationship between median and range of haemoglobin levels (g/dL) at baseline (*n* = 383) and at 5 months (*n* = 321) and 8 months (*n* = 332) after baseline treatment for the single-dose and double-dose Praziquantel treatments. The thick line within each box stands for the median haemoglobin value. The lower and upper edges of each box represent the 25th and 75th percentiles, respectively. The lower and upper whiskers represent the lower and upper values (range), respectively, excluding outliers.

The baseline mean haemoglobin levels in both treatment arms were compared with those at 5 months and 8 months after baseline treatment. In the single-dose Praziquantel treatment arm, the baseline mean haemoglobin level, 11.51 g/dL (95% CI: 11.32–11.70), did not differ significantly from that at 5 months after baseline treatment, 11.40 g/dL (95% CI: 11.19–11.62) (*p* > 0.05), but it was significantly smaller as compared to that at 8 months after baseline treatment, 13.10 g/dL (95% CI: 12.95–13.25) (*p* < 0.001). Likewise, in the double-dose Praziquantel treatment arm, the baseline mean haemoglobin level, 11.75 g/dL (95% CI: 11.60–11.91), did not differ significantly from that at 5 months after baseline treatment, 11.62 g/dL (95% CI: 11.40–11.85) (*p* > 0.05), but it was significantly smaller as compared to that at 8 months after baseline treatment, 13.09 g/dL (95% CI: 12.95–13.23) (*p* < 0.0001) (Figure 3). No significant difference was observed when mean haemoglobin levels at 8 months after baseline treatment were compared between the two treatment groups (*p* = 0.9374).

The increase in the mean haemoglobin levels at 8 months after baseline treatment resulted in a decrease in the prevalence of anaemia. The prevalence of anaemia at baseline, 29.43% (95% CI: 25.49%–33.38%), was compared with that 8 months after treatment, 3.84% (2.09%–5.63%). It was generally observed that the baseline prevalence of anaemia was significantly higher as compared to that at 8 months after treatment (*p* < 0.001). The prevalence of anaemia at baseline and that at 8 months after treatment were compared among individuals who received a single dose of Praziquantel and those who received two doses of Praziquantel three weeks apart. It was observed that, in both treatment arms, the prevalence of anaemia 8 months after baseline treatment was significantly lower when compared to that at baseline (*p* < 0.001).

## 4. Discussion

Praziquantel's restricted activity to adult worms and eggs may contribute to reduced efficacy of the drug and to raising population of adult parasites that have once been exposed to the drug and may possibly contribute to emergency of Praziquantel resistance [22]. Assessing alternative treatment regimen that would improve the drug's efficacy, thereby significantly hastening efforts to achieve transmission control, is of paramount significance. Therefore, this study intended to investigate the efficacy of single-dose and double-dose Praziquantel treatments on* S. mansoni* infection and its implication on the burden of undernutrition and anaemia among primary schoolchildren living in an endemic area in Rorya District, northwestern Tanzania.

In this study, we examined the impact of two repeated doses of 40 mg/kg Praziquantel administered 3 weeks apart compared to a standard single dose of 40 mg/kg with particular attention to cure rate and egg reduction rate (intensity of infection after treatment) and its effect on the burden of anaemia and undernutrition among study participants.

At 8 weeks after baseline treatment, we found significantly higher cure and egg reduction rates among individuals who were treated with double Praziquantel doses as compared to those treated with a single standard dose. 8 months after baseline treatment, we found that about 85% and 83% of those who were cured at 8 weeks after receiving a single treatment and two treatments, respectively, were reinfected. No significant difference was observed in the rates of reinfection at 5 months and 8 months after baseline treatment between the two treatment groups. It was further observed that, at 8 months after baseline treatment, there was no difference in the prevalence of stunting between the two treatment regimens. However, we noticed a significant increase in the prevalence of wasting among those on repeated dose compared to those on a single dose of Praziquantel. We further observed an increase in the mean haemoglobin levels at 8 months with no difference between the two arms.

The cure rate resulting from repeated Praziquantel treatments which is reported in this study is slightly higher compared to the upper margin of the possible cure rate resulting from single treatment (90%), while the cure rate resulting from single-dose standard treatment lies close to the lower margin of the recorded cure rate of single-dose Praziquantel treatment which is 60%–90% [5]. However, the cure rates reported in this study in both treatment arms are relatively higher than what was reported by Kabatereine (41.9% for single dose and 69.1% for double dose) and Tukahebwa (47.9% in single-dose and 69.7% in double-dose treatments) [36, 43]. This difference in the observed cure rates for the two treatment regimens in comparison with those reported in the previous studies could be due to differences in the timing of the second treatment, whereby in Kabatereine et al.'s study the two treatments were given 6 weeks apart and in Tukahebwa et al.'s work the two treatments were given 2 weeks apart and assessment was done at 6 weeks and/or 12 weeks and 9 weeks after treatment, respectively [36, 43], contrary to this study in which the two treatments were given at three weeks' interval and assessment was done at 8 weeks following baseline treatment. Also, it could possibly be due to the fact that the drug has not been intensively used in the study area for schistosomiasis mass chemotherapy. The relatively lower cure rate observed in the single-dose treatment arm as compared to repeated treatment suggests that since this is a* S. mansoni* endemic area [12], it is likely that during the baseline treatment some infected children had both mature worms and immature worms that are normally less sensitive to Praziquantel [22, 23]. This population of immature schistosomes that survived the first treatment, if left untreated, could result in adult parasites that are less sensitive to the drug. Administering a second treatment might result in killing those parasites that were immature at the time of the first treatment, resulting in improvement of the cure rate as seen in this study and delaying development of resistance to the drug. It was further observed that the superiority of the cure rates resulting from repeated Praziquantel treatment was observed across sex and village of residence, but this was not the case across age groups; we noted that there was no difference in cure rates among children aged 13–16 years who received single-dose and double-dose Praziquantel treatment. This observation could be attributed to the fact that this is the age at which highest prevalence is usually observed and their behaviour predisposes them to rapid reinfections following the first dose in such a way that when the second treatment was offered, infections acquired following the first dose were still at the age at which they were less sensitive to the second Praziquantel dose and therefore the lack of significant difference in cure rates in this age group was observed. This has also been reported as being a reason for low cure rate among children in this age group [44].

The primary objective for the currently used mass treatment programs in schistosomiasis endemic areas is morbidity reduction through reduction of the intensity of infection following treatment [36]. Double praziquantel doses resulted in a significantly higher egg reduction rate. Similar findings have also been reported in a different study [45]. The egg reduction rates reported in this study are within the recorded standard egg reduction rates of Praziquantel of over 80% or 90% [46, 47]. The relatively higher egg reduction resulting from double doses could, as stated earlier, be linked to the fact that, in areas where transmission intensity is very high, like an area in which this study was done [12], a single dose is not enough to kill all worms, particularly the immature worms; therefore administering a second dose resulted in killing more worms and eggs, resulting in the observed significantly higher cure and egg reduction rates [45]. In addition to this, the repeated-dose treatment in our study leads to a significantly lower mean egg intensity among those who were not cured at 8 weeks after baseline treatment. This could have been of value as far as morbidity reduction is concerned had it been sustained; however, in this study, no difference in geometric mean egg intensity between the two arms was observed at 5 months and 8 months after baseline treatment. This suggests that repeated treatments would not offer any added advantage on reducing* Schistosoma*-related morbidity in highly endemic areas, where infections with parasites at different developmental stages and rapid reinfections following treatment are a norm. To sustain the benefit of repeated treatments, treatment should be coupled with other control measures that will reduce the rate of reinfections following treatments such as behaviour change communication and sanitation [20]. This has also been reported in another longitudinal study, where treatments were done in different years and cure rates reported; it was observed that the year in which cure failures were the greatest was also the year in which prevalence of* S. mansoni* in snails was highest [48].

It has been reported that the rate of reinfection with schistosome parasites following Praziquantel treatment is faster with* Schistosoma mansoni* than with* Schistosoma haematobium* [25, 49–51]. Factors that determine the rate of reinfection with schistosome parasites have been said to include baseline infection intensity, schistosome species, and local ecology [25, 50, 52]. This study found overall prevalence of reinfection at 5 months and 8 months not to be statistically significantly different between the two treatment regimens, similar to what was also observed elsewhere [43]. This is likely a result of the high transmission intensity in the area where this study was conducted [12]. However, a significant difference in reinfection rate was observed when the analysis was based on village of residence. In Kibuyi village, a significant difference in reinfection rate was observed between the two treatments, with those receiving two treatments having significantly lower prevalence of reinfection at 8 months after baseline treatment. This observation could be a result of the difference in transmission intensity between the two villages, whereby Kibuyi has relatively lower transmission intensity as reported in our earlier report [12].

Intestinal schistosomiasis has been shown to contribute to the high prevalence of malnutrition and anaemia among children in developing world and improvement in nutritional status and haemoglobin levels has been reported following treatment with Praziquantel [53–56]. Although our previous study among study participants could not establish the relationship between nutritional status and anaemia with* S. mansoni* infection [34], we assessed the comparative implication of treating infected children with single-dose and repeated-dose Praziquantel treatment on the overall burden of anaemia and undernutrition among study participants. As an indicator of chronic nutritional insult, stunting rates at 8 months after baseline treatment did not differ significantly between the two treatment arms; however, there was a general decline in the prevalence of stunting when compared to baseline prevalence in both arms, a decline which of course this study fails to empirically link to treatment intervention due to lack of dietary information with regard to the study participants and the lack of a placebo control group. Surprisingly and contrary to stunting, the study observed that there was an increase in the overall rates of wasting following treatment, with the prevalence of wasting being significantly higher among children who were given repeated Praziquantel treatments. This observation relates to what was reported elsewhere demonstrating that* S. mansoni*-infected children were less likely to be wasted than their uninfected counterparts [57]. In this case, therefore, the slight improvement in linear growth as a result of treatment might have negatively affected weight- and height-based indices (BMIAZ) as reported in [57, 58] or the observed increase in the prevalence of wasting might have been confounded by acute dietary deficiency during or close to the time of the follow-up survey.

It has further been shown that* S. mansoni* infections contribute to the burden of anaemia among schoolchildren in endemic areas [59]. Although our earlier study in the study area could not associate* Schistosoma mansoni* infections with anaemia [34], we assessed the comparative implication of single-dose and repeated-dose Praziquantel treatments on the burden of anaemia among study participants. We found that there was a general increase in the mean haemoglobin levels among study participants in both treatment arms with ultimate significant decrease in the prevalence of anaemia following treatment among subjects in both treatment arms. Improvement in haemoglobin levels following Praziquantel treatment among* Schistosoma mansoni*-infected individuals has also been reported in other studies [56, 60, 61]. However, at 8 months after treatment, second dose of Praziquantel did not offer any added benefit in improvement of haemoglobin levels. This observation is likely to be due to the observed lack of difference in the prevalence of reinfection at 8 months, since morbidity to schistosomiasis correlates with the intensity and duration of infection [62].

We acknowledge that the relatively higher baseline arithmetic mean/geometric mean egg intensity among individuals who received two treatment doses might have resulted in underestimating the efficacy of the repeated treatments on* S. mansoni* and its possible implication on nutritional status and anaemia.

## 5. Conclusion and Recommendation

The present study found a significantly higher cure rate and egg reduction rates resulting from repeated-dose Praziquantel treatment three weeks apart as compared to a single standard dose at eight weeks after baseline treatment. However, besides the two treatment regimens resulting in significantly higher cure and egg reduction rates, the rates of reinfections among study subjects were almost equal between the two treatment arms, leading to the mean egg intensities becoming almost equal at 5 and 8 months after baseline treatment. To achieve reduction of transmission intensity and ultimately disease control in highly endemic areas, repeated treatments need to be coupled with other control measures such as behaviour change communication and improvement in water supplies and sanitation. The present study further noticed no difference in the prevalence of stunting between the two treatment regimens 8 months after baseline treatment. This could be a result of the short follow-up period; we therefore recommend studies that will have a longer follow-up period to assess the potential benefit of repeated treatments on nutritional status.

Significant increase in the mean haemoglobin levels following Praziquantel treatment among* Schistosoma mansoni*-infected individuals has also been reported in several studies [56, 60, 61]. Overall, there was a general significant increase in the mean haemoglobin levels among study subjects in our study with no difference between the two treatment arms. This highlights the usefulness of the currently used treatment regimen with the aim of controlling morbidity including anaemia. However, when the goal is to reduce transmission and ultimately achieve disease control, repeated-treatment regimen could offer a better benefit as compared to single-treatment regimen, particularly in areas where rapid reinfection occurs following treatment.

## Figures and Tables

**Table 1:** Baseline characteristics of study participants.

Characteristic	Single treatment *n* = 199	Repeated treatments *n* = 184	*p* value
Sex, female (%)	102 (51.26)	100 (54.35)	0.545^*∗*^
Mean age (95% CI)	11.05 (10.73–11.37)	11.14 (10.82–11.46)	0.6923^*∗∗*^
Mean Hb (95% CI)	11.51 (11.32–11.70)	11.75 (11.60–11.91)	0.055^*∗∗*^
GMI (95% CI), epg	152.98 (127.40–183.70)	203.00 (167.81–245.56)	**0.03**5^**∗∗**^
AMI (95% CI), epg	344.71 (261.13–428.30)	456.29 (341.32–571.26)	**0.04**7^†^
Mean height (cm) (95% CI)	134.55 (133.15–135.96)	134.57 (133.17–135.97)	0.9880^*∗∗*^
Mean weight (kg) (95% CI)	28.71 (27.87–29.54)	28.66 (27.80–29.51)	0.9316^*∗∗*^

Note: ^*∗*^chi-square test; ^*∗∗*^Student's *t*-test; ^†^Mann–Whitney *U* test.

**Table 2:** Cure rates of PZQ 40 mg/kg stratified by demographic characteristics and baseline infection intensity.

Characteristic	Treatment regimen				
	Single dose		Double dose		*p* value
	Treated (*N*)	Cured *n* (%(95% CI))	Treated (*N*)	Cured *n* (%(95% CI))	
Overall	182	125 (68.68 (61.90–75.46))	174	162 (93.10 (89.31–96.89))	<0.001
*Sex*					
Male	89	66 (74.16 (64.94–83.69))	81	78 (96.30 (92.13–100))	<0.001
Female	93	59 (63.44 (53.54–73.35))	93	84 (90.32 (84.24–96.40))	<0.001
*Village*					
Kibuyi	96	61 (63.54 (53.80–73.28))	85	82 (96.47 (92.49–100))	<0.001
Busanga	86	64 (74.42 (65.08–83.76))	89	80 (89.89 (83.54–96.23))	0.007
*Age (years)*					
6–9	38	19 (50.00 (33.63–66.37))	39	38 (97.44 (92.33–100))	<0.001
10–12	84	60 (71.43 (61.64–81.22))	87	81 (93.10 (87.71–98.49))	<0.001
13–16	60	46 (76.67 (65.75–87.58))	48	43 (89.58 (80.75–98.41))	0.080
*Intensity*					
Light	64	47 (73.44 (62.41–84.46))	49	46 (93.88 (87.02–100))	0.005
Moderate	86	57 (66.28 (56.15–76.41))	66	63 (95.45 (90.35–100))	<0.001
Heavy	32	21 (65.63 (48.68–82.57))	59	53 (89.83 (81.95–97.72))	0.005

*p* values are based on chi-square statistic.

**Table 3:** Reinfection with *S. mansoni* at 5 months and 8 months after treatment stratified by sex, village, and age.

Characteristic	5 months					8 months				
	1 × 40 mg/kg		2 × 40 mg/kg		*p* value	1 × 40 mg/kg		2 × 40 mg/kg		*p* value
	Cured at 8 weeks *N*	Reinfected at 5 months *n* (%)	Cured at 8 weeks *N*	Reinfected at 5 months *n* (%)		Cured at 8 weeks *N*	Reinfected at 8 months *n* (%)	Cured at 8 weeks *N*	Reinfected at 8 months *n* (%)	
Overall	112	74 (66.07)	150	102 (68.00)	0.742	121	100 (82.64)	148	114 (77.03)	0.256
*Sex*										
Male	60	43 (71.67)	69	45 (65.22)	0.433	65	55 (84.62)	71	59 (83.10)	0.810
Female	52	31 (59.62)	81	57 (70.37)	0.201	56	45 (80.36)	77	55 (71.43)	0.239
*Village*										
Kibuyi	58	36 (62.07)	72	38 (52.78)	0.288	67	61 (91.04)	78	59 (75.64)	**0.01**4^**∗**^
Busanga	54	38 (70.37)	78	64 (82.05)	0.115	54	39 (72.22)	70	55 (78.57)	0.413
*Age (years)*										
6–9	17	13 (76.47)	36	29 (80.56)	0.732	28	22 (78.57)	39	35 (89.74)	0.206
10–12	56	35 (62.50)	73	51 (69.86)	0.379	53	45 (84.91)	75	56 (74.67)	0.162
13–16	39	26 (66.67)	41	22 (53.66)	0.235	40	33 (82.50)	34	23 (67.65)	0.138

*∗* indicates statistical significance.

**Table 4:** Comparison of proportion of people with stunting and wasting at baseline and at 8 months after treatment.

Morbidity	*n*	Prevalence at baseline % (95% CI)		Prevalence at 8 months % (95% CI)	*p* value
*Stunting*					
Overall	383	40.21 (35.30–45.12)	336	36.31 (31.17–41.45)	0.2833
*Treatment arm*					
1 × 40 mg/kg	199	38.19 (31.44–44.94)	175	37.14 (29.98–44.30)	0.8344
2 × 40 mg/kg	184	42.39 (35.25–49.53)	161	35.40 (28.01–42.79)	0.1844
*Wasting*					
Overall	383	14.10 (10.61–17.59)	336	24.40 (19.81–28.99)	<0.001
*Treatment arm*					
1 × 40 mg/kg	199	15.58 (10.54–20.62)	175	22.29 (16.12–28.45)	0.0970
2 × 40 mg/kg	184	12.50 (7.72–17.28)	161	26.71 (19.87–33.54)	0.0008

## Abbreviations

NIMR: National Institute for Medical Research
SSA: Sub-Saharan Africa
epg: Eggs per gram of faeces
GM: Geometric mean
MDA: Mass drug administration.
